# Genome-Wide Association Study for Levels of Total Serum IgE Identifies *HLA-C* in a Japanese Population

**DOI:** 10.1371/journal.pone.0080941

**Published:** 2013-12-04

**Authors:** Yohei Yatagai, Tohru Sakamoto, Hironori Masuko, Yoshiko Kaneko, Hideyasu Yamada, Hiroaki Iijima, Takashi Naito, Emiko Noguchi, Tomomitsu Hirota, Mayumi Tamari, Yoshimasa Imoto, Takahiro Tokunaga, Shigeharu Fujieda, Satoshi Konno, Masaharu Nishimura, Nobuyuki Hizawa

**Affiliations:** 1 Department of Pulmonary Medicine, Faculty of Medicine, University of Tsukuba, Ibaraki, Japan; 2 Tsukuba Medical Center, Ibaraki, Japan; 3 Department of Medical Genetics, Faculty of Medicine, University of Tsukuba, Ibaraki, Japan; 4 Laboratory for Respiratory Diseases, Center for Genomic Medicine, the Institute of Physical and Chemical Research (RIKEN), Kanagawa, Japan; 5 Department of Otorhinolaryngology-Head and Neck Surgery, Faculty of Medicine, University of Fukui, Fukui, Japan; 6 First Department of Medicine, School of Medicine, Hokkaido University, Hokkaido, Japan; National Cancer Institute, National Institutes of Health, United States of America

## Abstract

Most of the previously reported loci for total immunoglobulin E (IgE) levels are related to Th2 cell-dependent pathways. We undertook a genome-wide association study (GWAS) to identify genetic loci responsible for IgE regulation. A total of 479,940 single nucleotide polymorphisms (SNPs) were tested for association with total serum IgE levels in 1180 Japanese adults. Fine-mapping with SNP imputation demonstrated 6 candidate regions: the *PYHIN1/IFI16,* MHC classes I and II, *LEMD2, GRAMD1B,* and chr13∶60576338 regions. Replication of these candidate loci in each region was assessed in 2 independent Japanese cohorts (n = 1110 and 1364, respectively). SNP rs3130941 in the *HLA-C* region was consistently associated with total IgE levels in 3 independent populations, and the meta-analysis yielded genome-wide significance (*P = *1.07×10^−10^). Using our GWAS results, we also assessed the reproducibility of previously reported gene associations with total IgE levels. Nine of 32 candidate genes identified by a literature search were associated with total IgE levels after correction for multiple testing. Our findings demonstrate that SNPs in the *HLA-C* region are strongly associated with total serum IgE levels in the Japanese population and that some of the previously reported genetic associations are replicated across ethnic groups.

## Introduction

Immunoglobulin E (IgE) is a class of antibodies that has an important role in the development of Th2 cell-mediated allergic inflammatory diseases such as asthma, allergic rhinitis, and atopic dermatitis. In atopic individuals, exposure to allergens results in Th2 cell-dependent stimulation of the immune response that causes production of IgE. Recent advances in the understanding of allergen sensitization have also revealed the sentinel role of innate immune mechanisms involved in the development of allergic diseases [1], [2].

Twin and family studies have shown that genetic factors are important for total serum IgE levels [3], [4] and account for about 36% to 78% heritability of its levels [3], [5]. Furthermore, it has been demonstrated that total serum IgE levels are mainly determined by genetic factors that are independent of antigen-specific IgE levels or atopic status [4], [6], [7]. Asthma affection status is known to be related to total serum IgE levels even after adjustment for atopic status [8], [9].

Thus far, a number of candidate gene association studies for total serum IgE levels have demonstrated many polymorphisms in genetic regions related to the Th2 cell-dependent pathways. Recently, 4 genome-wide association studies (GWASs) of total serum IgE levels in independent populations have revealed additional genetic loci, such as *TBX18* and *SOBP*, which seem to be unrelated to the Th2 cell-dependent pathways [10]–[13]. Thus, because GWASs are unbiased by investigator preconceptions, they have the potential of providing new insights into the mechanism of IgE regulation and may be able to clarify unexpected IgE-related genetic loci.

Three of the 4 GWASs of total IgE levels reported so far were conducted solely in populations of European ancestry, and the fourth of those studies also included African-American and Latino populations. In contrast, 2 GWASs recently performed in Asian populations did not identify any loci significantly associated with total serum IgE levels [14], [15]. In genetic association studies, replication of the initial findings in different ethnic groups is important to clarify the relevance of the findings.

Here, we performed a GWAS of total serum IgE levels in a Japanese population and a replication analysis in 2 independent Japanese cohorts that was followed by a meta-analysis. In addition, we validated previously reported gene associations with total serum IgE levels using our GWAS data.

## Results

### Study Flow Chart

A flow chart outlining the steps of this study is shown in Figure S1.

### Characteristics of the Study Cohorts

The characteristics of the original GWAS cohort and the replication cohorts are provided in Table 1. The ratio of female participants was higher in the Fukui cohort than in the Tsukuba and Hokkaido cohorts. The Tsukuba and Hokkaido cohorts included more asthmatic patients than did the Fukui cohort. Age, sex, asthma affection status, atopic status, and IgE levels differed significantly among the cohorts.

**Table 1:** Characteristics of the study cohorts.

	Tsukuba cohort (n = 1180)	Hokkaido cohort (n = 1110)	Fukui cohort (n = 1364)
Age, y (SD)	50.3 (10.4)	44.6 (16.0)	32.2 (9.8)
Female sex	55.3%	49.3%	67.0%
Smoking status			
Current smoker	16.2%	26.1%	NA
Ex-smoker	17.5%	16.7%	NA
Never smoker	66.2%	57.2%	NA
Asthma	18.1%	44.2%	6.5%
Atopy			
Atopic	56.6%	53.7%	67.2%
Nonatopic	40.5%	29.5%	30.5%
Unknown	2.9%	16.8%	2.3%
Log_10_ [total serum IgE] (SD)	1.82 (0.60)	2.08 (0.69)	1.91 (0.62)

NA = not applicable; SD = standard deviation.

### GWAS and Replication Analyses

**Figure 1:**
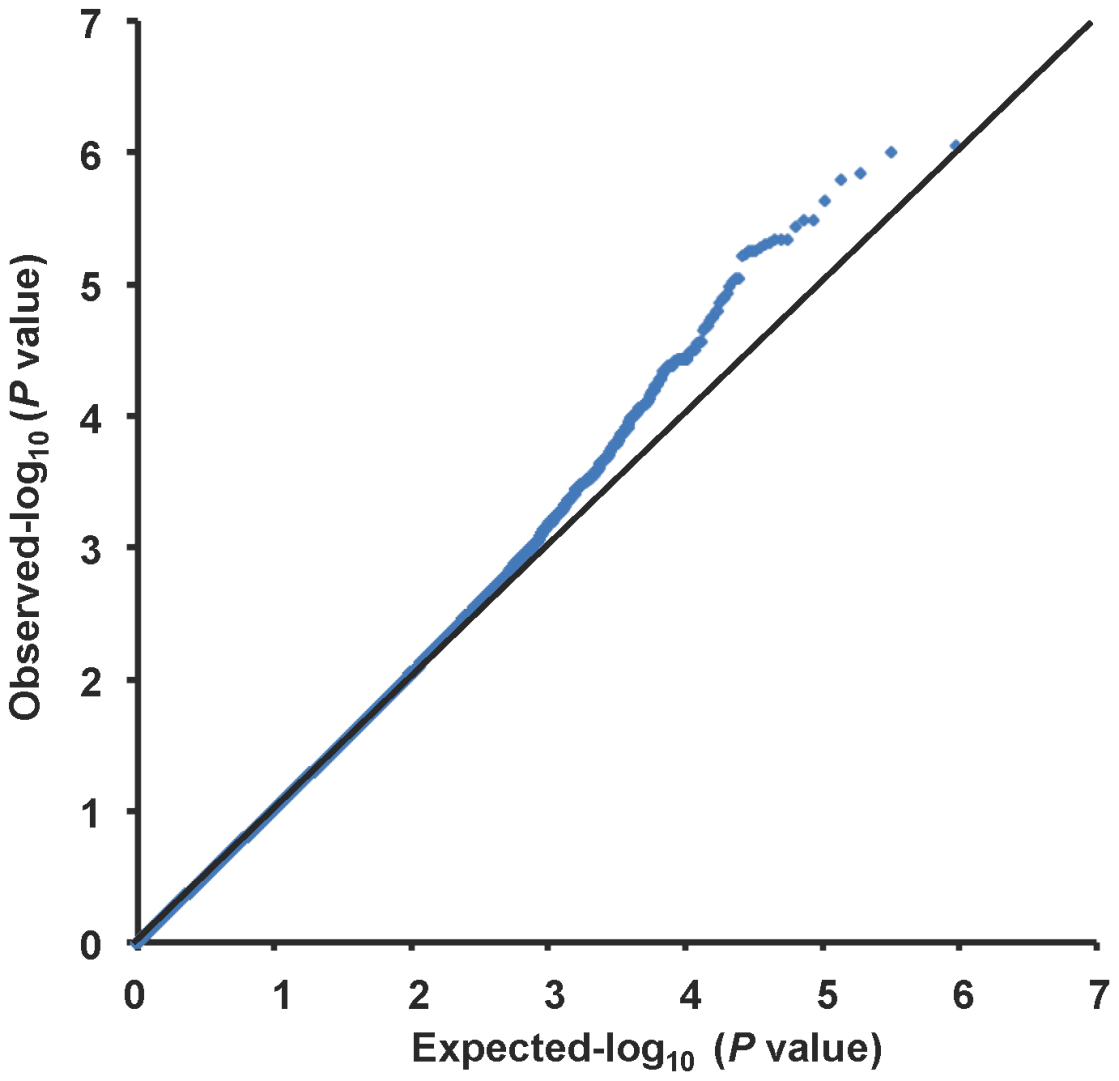
Quantile-quantile (Q-Q) plot of observed versus expected *P* values of the GWAS results. The straight line in the Q-Q plot indicates the distribution of SNPs under the null hypothesis.

**Figure 2:**
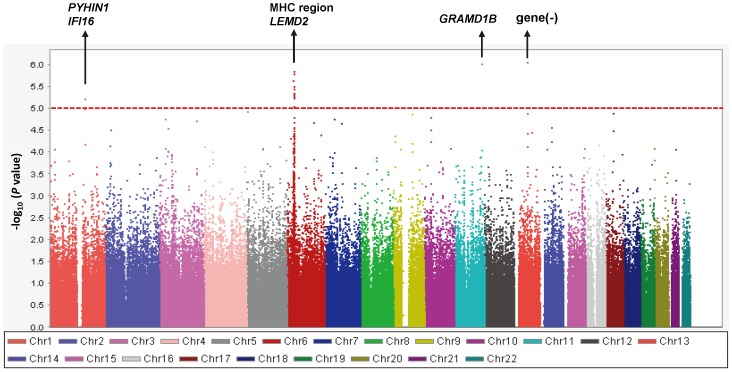
Manhattan plots of −log_10_ (*P* value) for association of 479,940 SNPs with total IgE levels. Linear regression models adjusted for age, sex, smoking status (never, ex-, or current smoker), pack-year group (0, 0–10, or >10), and asthma affection status were performed. The red line shows the threshold (*P* = 1×10^−5^) for selection of genomic regions for further analysis including SNP imputation.

**Figure 3:**
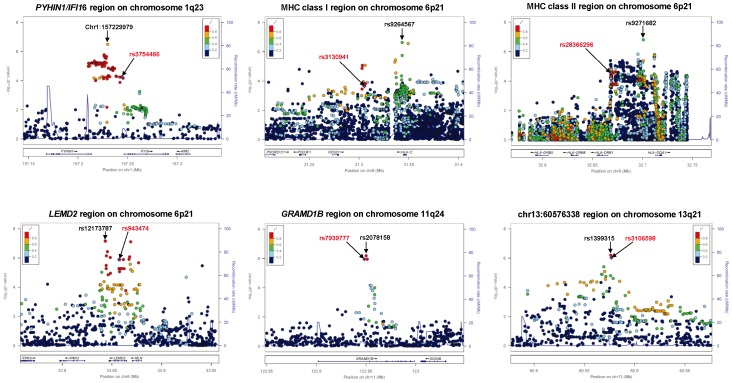
Fine-mapping identification of 6 candidate genomic regions. Plots show the association results of both genotyped and imputed SNPs in the primary GWAS cohort. The most strongly associated SNPs (black letters) in the GWAS and the SNPs used for the replication studies (red letters) are indicated by arrows. The color of each circle reflects the LD (r^2^) between a particular SNP and the SNP used for the replication studies.

A quantile-quantile plot is shown in Figure 1. The genomic inflation factor of 1.018 indicated a low possibility of false-positive associations resulting from population stratification. A Manhattan plot of the GWAS (Figure 2) showed no SNPs reaching the genome-wide significance threshold of 5.0×10^−8^. We focused on 4 distinct chromosomal regions in which *P* values were less than 1.0×10^−5^: chromosomes 1q23, 6p21, 11q24, and 13q21. Genotypes were imputed to determine the contribution of untyped SNPs to total IgE levels in these regions. Fine-mapping coupled with the imputed SNPs identified 6 candidate genomic regions (Figure 3): the *PYHIN1/IFI16* region on chromosome 1q23.1 (chr1∶157229979; *P* = 3.19×10^−7^), the MHC class I and II regions on chromosome 6p21.3 (rs9264567, *P* = 2.33×10^−7^ and rs9271682, *P* = 1.55×10^−7^, respectively), the *LEMD2* region on 6p21.31 (rs12173787, *P* = 7.03×10^−8^), the *GRAMD1B* region on chromosome 11q24.1 (rs2078158, *P* = 6.57×10^−7^), and the chr13∶60576338 region on chromosome 13q21.31 (rs1399315, *P* = 6.40×10^−7^). For each candidate region, we conducted a replication study using validated ready-to-use TaqMan® SNP Genotyping assays. The SNPs available for the replication studies are shown in Figure 3. Table 2 shows the association of these SNPs with total serum IgE levels in the primary Tsukuba cohort and the 2 replication cohorts. The association of rs3130941 in the MHC class I region was consistently replicated in both the Hokkaido and the Fukui cohort. Meta-analysis of the primary and the 2 replication cohorts demonstrated that rs3130941 in the MHC class I region reached the level of genome-wide significance at 1.07×10^−10^. Rs28366296 in the MHC class II region was replicated in the Hokkaido cohort only. Rs7939777 in the *GRAMD1B* region was replicated in the Fukui cohort only. As for rs7939777, a meta-analysis using the Tsukuba and Fukui cohorts yielded a *P* value of 3.35×10^−10^.

**Table 2:** Replication studies and meta-analysis.

		Tsukuba cohort						Hokkaidocohort	FukuiCohort	Meta-analysis
		Top SNP								
Chromosome	Gene	SNP forreplicationstudy	*P* value	LD withtop SNP(r^2^)	MAF	Minorallele	β	*P* value	*P* value	*P* value
1q23.1	*PYHIN1/IFI16*	chr1∶157229979	3.19E-07	1	0.16	G	−0.168			
		rs3754466	5.65E-05	0.637	0.20	C	−0.119	0.244	0.222	0.378
6p21.3	MHC class I	rs9264567	2.33E-07	1	0.28	A	0.132			
		rs3130941	2.28E-04	0.549	0.25	C	0.098	5.03E-04	6.12E-05	1.07E-10
6p21.3	MHC class II	rs9271682	1.55E-07	1	0.48	A	0.127			
		rs28366296	2.67E-05	0.608	0.39	A	−0.098	4.15E-02	5.44E-02	2.17E-05
6p21.31	*LEMD2*	rs12173787	7.03E-08	1	0.17	G	0.161			
		rs943474	1.30E-06	0.786	0.16	G	0.157	5.56E-02	0.308	2.29E-02
11q24.1	*GRAMD1B*	rs2078158	6.57E-07	1	0.41	A	−0.118			
		rs7939777	1.13E-06	0.943	0.42	C	−0.114	7.63E-02*	7.58E-05	0.263
13q21.31	none	rs1399315	6.40E-07	1	0.48	C	0.114			
		rs3106598	9.51E-07	0.997	0.48	G	0.112	0.817	0.813	0.326

* The direction of the effect was opposite to that of the Tsukuba cohort.

LD = linkage disequilibrium; MAF = minor allele frequency.

When we repeated the meta-analyses by studying nonasthmatic healthy individuals only (n = 2861) or by adding atopic sensitization as an additional covariate, we confirmed the association between rs3130941 in the MHC class I region and levels of total serum IgE at genome-wide significance (Table S1 and Table S2).

### Validation of Previous Genetic Associations

The PubMed search identified 448 related publications. After screening of the titles, abstracts, and text, 156 eligible publications were selected. Screening of the references of those publications identified 33 additional relevant publications. From the 189 selected publications, we found 32 candidate genes associated with total serum IgE levels; 25 of those genes were reported in 3 or more candidate gene association studies, and 7 (*DARC*, *HLA-A*, *HLA-DQA2*, *HLA-G, RAD50, SOBP, and TBX18*) were reported in at least 1 GWAS.

Table 3 shows the SNP most significantly associated with total IgE levels in our GWAS data of each candidate gene on an autosomal chromosome. Nominal *P* values were less than 0.05 for 17 of the 32 candidate genes, including *HLA-C*. After corrections for multiple testing using SNPSpD software [16], the associations of 9 genes with total IgE levels remained significant. The strongest associations were detected at the genes in the MHC class I/II regions, including *LTA* on chromosome 6p21.3, although none of the top SNPs reached genome-wide significance. Previously reported polymorphisms associated with total serum IgE levels are shown in Table S3.

**Table 3:** Top SNPs with the strongest statistical evidence of association with total serum IgE levels.

Gene	Chromosome	Number of SNPs	SNP	Nominal *P* value	Corrected *P* value*
*ADAM33*	20	12	rs2853215	1.66E-02	0.150
*ADRB2*	5	17	rs17640574	7.27E-02	0.728
*CCL11*	17	7	rs6505403	0.225	0.898
*CD14*	5	8	rs3822356	0.719	1
*CMA1*	14	9	rs1956932	0.179	1
*CTLA4*	2	3	rs231726	1.21E-02	2.31E-02
*DARC*	1	3	rs863002	0.394	0.789
*FCER1A*	1	9	rs2427824	3.06E-02	0.184
*FLG*	1	3	rs3126085	0.288	0.577
*GSTP1*	11	3	rs614080	0.766	1
*HLA-A*	6	2	rs2734959	6.64E-03	1.33E-02
*HLA-C*	6	26	rs3132486	1.50E-04	1.88E-03
*HLA-DQA2*	6	24	rs17500468	7.94E-05	8.06E-04
*HLA-DQB1*	6	11	rs2647025	7.48E-05	3.74E-04
*HLA-DRB1*	6	66	rs35465556	8.51E-05	1.83E-03
*HLA-G*	6	21	rs1633053	7.58E-03	4.56E-02
*IFNG*	12	4	rs3181032	3.56E-02	0.104
*IL10*	1	8	rs1800896	0.110	0.660
*IL13*	5	4	rs1295686‡	7.41E-02	0.216
*IL4*	5	5	rs2243288	2.08E-03	8.34E-03
*IL4R*	16	23	rs4787948	0.219	1
*LTA*	6	12	rs2857709	4.24E-05	2.97E-04
*MS4A2*	11	4	rs574700	0.201	0.602
*NOD2*	16	6	rs7194886	0.156	0.468
*NOS1*	12	45	rs4767535	8.60E-03	0.235
*NPSR1*	7	66	rs323928	3.29E-02	0.867
*RAD50*	5	7	rs17772583	1.82E-02	7.29E-02
*SOBP*	6	41	rs3734747	5.43E-03	0.135
*STAT6*	12	7	rs841718	0.171	0.854
*TBX18*	6	5	rs2015519	3.71E-02	0.146
*TLR2*	4	4	rs7656411	0.328	0.983
*TNF*	6	11	rs2844484	7.96E-02	0.478

* The significance level was corrected for multiple testing using the SNPSpD program [16].

‡ SNP previously reported for association with total serum IgE (the direction of the effect was the same).

## Discussion

To the best of our knowledge, ours is the first GWAS that demonstrates positive results for levels of total serum IgE in an Asian population. In our primary GWAS cohort, fine-mapping using the imputed SNPs on chromosome 6p revealed 3 independent peaks: the MHC class I, MHC class II, and *LEMD2* regions (Figure S2). In the meta-analysis, rs3130941 in the MHC class I region reached levels of genome-wide significance. This finding was not significantly influenced by the presence or absence of asthma or atopy. Rs3130941 is located between *HLA-C* and *HCG27* (HLA complex group 27) (Figure 3). In the MHC class I region, 4 genes have been previously reported to be associated with total serum IgE levels: *HLA-A* (rs2517754, rs2571391), *HLA-G* (rs2523809), *LTA* (rs909253), and *TNF* (rs1800629, rs361525, rs1800630) [12], [17]–[22]. Linkage disequilibrium (LD) between rs3130941 and each of these SNPs estimated by r^2^ in our population was very weak (Table S4, Figure S3). Furthermore, the association of rs3130941 with total IgE levels was not influenced by inclusion of each of these SNPs in the statistical model as a covariate (Table S4). Therefore, rs3130941 in the *HLA-C* region is associated with total serum IgE levels independent of the genetic influence of *HLA-A*, *HLA-G*, *LTA,* or *TNF*. With respect to the functional consequence of rs3130941, the GENEVAR database (http://www.sanger.ac.uk/humgen/genevar/) [23] revealed that rs3094609 and rs3130931, both of which are in weak LD with rs3130941 (r^2^ = 0.566 and 0.324, respectively), are significantly associated with expression levels of *HLA-C* mRNA (*P* = 0.0495 and 0.00521, respectively), suggesting that rs3130941 may also be related to *HLA-C* expression.

The MHC class II region is another candidate for regulation of IgE levels because MHC class II molecules are importantly involved in antigen-specific IgE synthesis [24]. Three genes in the MHC class II region have been reported to be associated with total IgE levels: *HLA-DQA2*, *HLA-DQB1,* and *HLA-DRB1*. In our primary GWAS, associations of SNPs in these genes were replicated (Table 3). Because the MHC class I and II regions are in very close proximity on chromosome 6p21.3, we examined whether the genetic impact of rs3130941 in the MHC class I region was influenced by rs28366296 in the MHC class II region. Although both rs3130941 and rs28366296 showed a strong association with total IgE levels in our study, the LD between these 2 SNPs was weak (Table S4) and a linear regression model including these 2 SNPs showed that each genetic association maintained significance after controlling for the effect of each of the remaining SNPs (Table S4). Therefore, we believe that the association of rs3130941 with total serum IgE levels is independent of the effects of MHC class II genes.

A set of specific infections that strongly promote Th1 and natural killer (NK) cells likely has the potential to inhibit atopic disorder by repression of Th2 immunity. Among Japanese schoolchildren, positive tuberculin responses predicted a lower incidence of asthma, lower serum IgE levels, and biased Th1 cytokine profiles [25]. MHC class I-restricted CD8 T cells collaborate with CD4 Th1 cells to invoke Th1-type immunity, thereby counteracting CD4 Th2 cells, which results in inhibition of IgE production [26], [27]. HLA-C molecules also modulate NK cell function [28]. NK cytotoxicity is negatively controlled by inhibitory receptors, such as human killer cell Ig-like receptors (KIRs) specific for HLA-B and HLA-C [29]. Accordingly, as an intrinsic abnormality, impaired HLA-C-mediated triggering of protective immunity to microbial exposure would predispose an individual to increased levels of total serum IgE as a principal determinant of allergy.

Although localizing the causal effects of the genes within the MHC region has been limited by the complexity and strong LD of this region, the genetic association between the MHC region and several immune and inflammatory diseases has been among the most robust. The *HLA-C* region has been associated particularly with Behçet disease, psoriasis, and sarcoidosis [30]–[32]. Of note, dysregulation of IgE has been reported in these diseases [33]–[35], which may also imply that *HLA-C* is involved in the genetic regulation of total IgE levels. Finally, it is also interesting to note that 1 locus (rs9266772) near *HLA-C* and *MICA* has been recently identified as one of the loci of allergy-specific susceptibility [36].

The meta-analysis of rs7939777 in *GRAMD1B* using the Tsukuba and Fukui cohorts yielded a *P* value of 3.35×10^−10^. Interestingly, although no studies demonstrated a relationship between *GRAMD1B* and serum IgE levels, a GWAS has identified a SNP near *GRAMD1B* on chromosome 11q24.1 associated with chronic lymphocytic leukemia (CLL) [37]. CLL is characterized by coexpression of CD19 and CD23 coupled with low levels of surface immunoglobulins [38]. CD23, also known as FCER2, is a low-affinity receptor for IgE and important for regulation of serum IgE levels. In addition, total serum IgE levels are inversely associated with risk of CLL [39]. Accordingly, polymorphisms of *GRAMD1B* could be related to regulation of total serum IgE levels.

Although the SNP in the *PYHIN1/IFI16* region did not reach genome-wide significance in the primary GWAS (*P* = 3.19×10^−7^), a recent meta-analysis of GWASs of asthma has identified a SNP in *PYHIN1* in populations of African descent [40]. PYHIN1 and IFI16 have recently emerged as sensors of microbial DNA [41], and the innate immune response relies on the ability of immune cells to detect the presence of infection through these germline-encoded pattern recognition receptors. Given that environments with a wide range of microbial exposures are associated with protection from childhood asthma and atopy in proportion to their level of exposure to bacterial and fungal microbes [42], *PYHIN1* and *IFI16* deserve further attention as candidate genes for association with asthma and atopy.

We have here tried to validate previously reported gene associations with IgE regulation. Among 32 autosomal genes, which were previously identified mainly in European populations, we found that 9 (28.1%) were replicated in a Japanese population after correction for multiple testing, indicating that some of the candidate genes for association with total serum IgE levels are effective across ethnic groups. Heterogeneity seems to exist in the genetic factors for total serum IgE levels among different ethnic groups [13], [43]. For SNPs that were not replicated in our study, causal SNPs for IgE regulation or SNPs tightly in LD with the causal SNPs may not exist in the regions analyzed in the Japanese population. Alternatively, our study sample size may have not have provided a significant power to detect the associations due to low minor allele frequencies of the true causal SNPs.

In terms of the limitations of this study, because we chose only 1 SNP for each region to replicate the original findings in the discovery cohort, we cannot exclude the possibility that we have missed true functional genetic variants in the replication cohorts. In addition, we observed many differences in the population characteristics of the discovery cohort and of the replication cohorts, including in the proportion of asthmatic patients and levels of total serum IgE. These differences might have affected our results, especially because the genetic background for increased levels of total IgE may differ in nonasthmatic healthy individuals and in asthmatic patients [44]. Nevertheless, analyses excluding asthmatic patients produced similar results with genome-wide significance for the *HLA-C* region, indicating the robustness of our findings.

As the mechanisms mediating the risk conferred by the *HLA-C* region remains to be found, future studies will identify the causal genes/variants within the susceptibility loci associated with levels of total serum IgE by fine-mapping and by investigating the biological link between rs3130941/HLA-C and regulation of IgE production.

In summary, we performed a GWAS showing positive results for total serum IgE levels for the first time in an Asian population. Association of a SNP in the *HLA-C* region with total serum IgE levels reached genome-wide significance in our meta-analysis involving a total of 3654 Japanese adults. We also demonstrated that some of the previously reported genetic associations with total serum IgE levels were replicated across ethnicities.

## Materials and Methods

### Ethical Statement

This study was approved by the Human Genome Analysis and Epidemiology Research Ethics Committee of the University of Tsukuba and by the Human Genome/Gene Analysis Research Ethics Review Committees of the Tsukuba Medical Center, RIKEN, the Hokkaido University School of Medicine, and the University of Fukui. Written informed consent was obtained from each participant in accordance with institutional requirements and the principles of the Declaration of Helsinki.

### Study Participants

The discovery cohort (Tsukuba cohort) consisted of 1180 individuals of Japanese ethnicity (967 healthy volunteers and 213 patients with asthma). The healthy volunteers without pulmonary diseases such as asthma and COPD were originally recruited for a genetic study of pulmonary function from the general population who visited the Tsukuba Medical Center for an annual health checkup [45]. All the participants were asked about their respiratory health, medical history, lifestyle, and exposure to environmental irritants (eg, cigarette smoke, allergens, and air pollution) and underwent heart and lung auscultation. The patients with asthma were recruited for genetic analysis of asthma from the Tsukuba University Hospital and its affiliated hospitals [46]. Asthma was diagnosed by pulmonary physicians according to the American Thoracic Society criteria as previously described [47]. Specific serum IgE antibody was measured for both the healthy and the asthmatic groups with the multiple allergen simultaneous test (MAST)-26 chemiluminescent assay systems (Hitachi Chemical Company, Tokyo, Japan) [48]. Atopy was assessed by measurement of specific IgE responsiveness to 14 common inhaled allergens including *Dermatophagoides farinae*, grass pollens, animal dander, and molds. We defined atopy as a positive response (>4.40 lumicount) to at least 1 of the 14 allergens.

To replicate our findings in the discovery cohort, we analyzed 2 independent Japanese cohorts. The first replication cohort (Hokkaido cohort) comprised 619 healthy volunteers and 491 asthmatic patients from the Hokkaido University Hospital and its affiliated hospitals. This population was originally recruited for a case-control genetic association study searching for susceptibility genes to asthma and atopy [49]. Serum-specific IgE to *Dermatophagoides species*, molds, pollen, and animal dander was measured by a radioallergosorbent test (RAST). Atopy was defined as a positive response (>0.70 UA/mL) to at least 1 of these allergens.

The second replication cohort (Fukui cohort) comprised 1275 healthy volunteers and 89 asthmatic patients. This population was originally recruited from workers and students of the University of Fukui for a study of the genetic epidemiology of allergic rhinitis [50]. Serum IgE antibody specific to Japanese cedar, house dust, orchard grass, ragweed mix, *Candida species,* or *Aspergillus species* was measured by RAST. Atopy was defined as a positive response (>0.70 UA/mL) to at least 1 of these allergens.

### Genotyping

Genomic DNA was extracted from peripheral blood samples of all participants by an automated DNA extraction system (QuickGene-610L; Fujifilm, Tokyo, Japan). Genotyping of the Tsukuba cohort was carried out using the Illumina HumanHap550v3 BeadChip (Illumina, San Diego, CA, USA) for the healthy volunteers and the HumanHap610-Quad BeadChip for the asthmatic patients. The concordance rate between the genotypes determined by the Illumina HumanHap550v3 BeadChip and the Illumina HumanHap610-Quad BeadChip among 182 duplicated samples was 0.99998 [46]. Quality control checks for the SNPs were performed separately using PLINK version 1.07 software [51]. None of the healthy volunteers or the asthmatic patients were removed owing to a call rate for autosomal SNPs of <0.02. SNPs with a missing genotype rate >0.01, minor allele frequency <0.01, or Hardy-Weinberg equilibrium *P* value <1.0×10^−6^ were excluded, leaving 479,940 SNPs that were common to the 2 arrays for analysis. Raw data is available upon request.

Genotyping accuracy on the X chromosome is often lower than that on other chromosomes because of difficulties involving clustering algorithms, higher frequencies of chromosome anomalies, and more missing data on X chromosome variants [52]. Genotyping of the pseudoautosomal region shared with the Y chromosome and hemizygous males can also be problematic. These analytic complexities could reduce the power of X chromosome analyses, making detection of reliable associations difficult. Therefore, in the current study, we decided to exclude the X chromosome from the analysis.

We imputed the genotypes of missing SNPs by using MACH version 1.0 software [53] to improve the resolution of candidate regions identified as associated with total IgE levels at *P* values <1×10^−5^. MACH employs a Markov chain algorithm and imputes missing genotypes by taking phased haplotypes as templates. We used 1000 Genomes Project data of Asian origin (JPT+CHB) (http://www.sph.umich.edu/csg/abecasis/MACH/download/1000G-2010-06.html) as the reference panel. To evaluate missing genotypes, we used 50 iterations of the Markov sampler to ensure reliable results.

To obtain the genomic inflation factor, we performed the multidimensional scaling (MDS) method using PLINK version 1.07 software. The MDS method is widely used in stratification methods, matching cases to controls based on genotype information (identity-by-state), resulting in discrete strata of individuals that can be analyzed using the Cochran–Mantel–Haenszel test [51].

For replication analyses of the original GWAS data, to obtain high-confidence results, we selected SNPs that are available in the ready-to-use predesigned TaqMan® SNP Genotyping assays (Applied Biosystems, Foster City, CA, USA) in each candidate region that satisfied the following conditions in the discovery Tsukuba cohort: (1) in strongest LD with the SNP most significantly associated with total IgE levels and (2) minor allele frequency >0.15. All assays are quality control tested using a mass spectrophotometer to verify sequence and yield. All assays have 1 VIC® and 1 FAM™ dye-labeled probe and 2 target-specific primers and undergo bioinformatics evaluation of target SNP sequences.

### Validation of Association of Previously Reported Genes

A literature search was conducted in PubMed of publications up to June 1, 2013 on genetic association studies of total serum IgE levels. Keywords in the search strategy were (“IgE level” or “IgE concentration” or “serum IgE”) and (“polymorphism” or “SNP” or “genetics”) and (“association”). The search was restricted to human studies written in English. We reviewed the titles, abstracts, and texts of the publications to identify positive genetic association studies. Review articles and studies analyzing antigen-specific IgE production were excluded, as were studies using linkage analysis and transmission disequilibrium tests. We selected only genetic association studies. The references of the collected articles were also screened to find additional matching studies. From the retrieved publications, we selected eligible genes that were reported in 3 or more independent association studies or demonstrated by at least 1 GWAS so that we could as far as possible exclude potentially false-positive findings.

Because LD structures may be quite different between Japanese and Caucasian populations, we attempted gene-level replication instead of SNP-level replication. From the primary GWAS data, we chose the SNP with the strongest statistical evidence in a region extending +/−10 kilobases (kb) from each literature-selected candidate gene. The significance level was corrected for multiple testing using the SNPSpD program [16], which corrects for multiple testing of SNPs in LD with each other on the basis of the spectral decomposition of matrices of pairwise LD between SNPs. This method provides a useful alternative to more computationally intensive permutation tests.

### Statistical Analysis

In the primary GWAS cohort, associations of genotypes of all the SNPs with log-transformed (base 10) levels of total serum IgE were analyzed by multiple linear regression models in PLINK version 1.07. Because total serum IgE levels are influenced by age, sex, smoking status, and asthma affection status [8], [54], the original GWAS of the total serum IgE levels in the current study was adjusted according to these variables. Quantile-quantile plots and genomic inflation factors were calculated in PLINK version 1.07. In the replication studies, the associations were examined by the same methods in the Hokkaido cohort. As smoking behavior was not available in the Fukui cohort, the associations were adjusted only for age, sex, and asthma affection status in this cohort. Replication was declared only if *P*<0.05 and the direction of the effect was the same as in the primary GWAS. Combined analysis of the primary GWAS with the replication studies was performed by the basic meta-analysis function in PLINK version 1.07. Random-effect meta-analysis *P* values were estimated. We used the Haploview 4.2 program [55] to analyze the LD values between SNPs.

## Supporting Information

Figure S1 **Study flow chart.** GWAS for total IgE levels was performed, followed by replication studies and meta-analysis. Validation of previously reported genes for IgE was also conducted using the GWAS data.
(TIFF)

Figure S2 **Fine-mapping association plots on chromosome 6p21.** Three peaks are identified: the MHC class I, MHC class II, and *LEMD2* regions.
(TIFF)

Figure S3 **Fine-mapping association plots in the MHC class I region.** The color of each circle reflects the LD (r^2^) between a particular SNP and rs3130941 indicated as a purple diamond.
(TIFF)

Table S1 Results of meta-analysis for nonasthmatic healthy individuals only.
(DOCX)

Table S2 Results of meta-analysis after inclusion of atopic status as a covariate.
(DOCX)

Table S3 Previously reported polymorphisms significantly associated with total serum IgE.
(DOCX)

Table S4 Genetic influences of SNPs in the MHC class I/II regions on the association between rs3130941 and total IgE levels.
(DOCX)
