# Supplementary figures and images for: Genome-Wide Association Study for Levels of Total Serum IgE Identifies HLA-C in a Japanese Population
Source: PLoS One. 2013 Dec 4;8(12):e80941. doi: 10.1371/journal.pone.0080941 (PMC3851760; doi:10.1371/journal.pone.0080941)

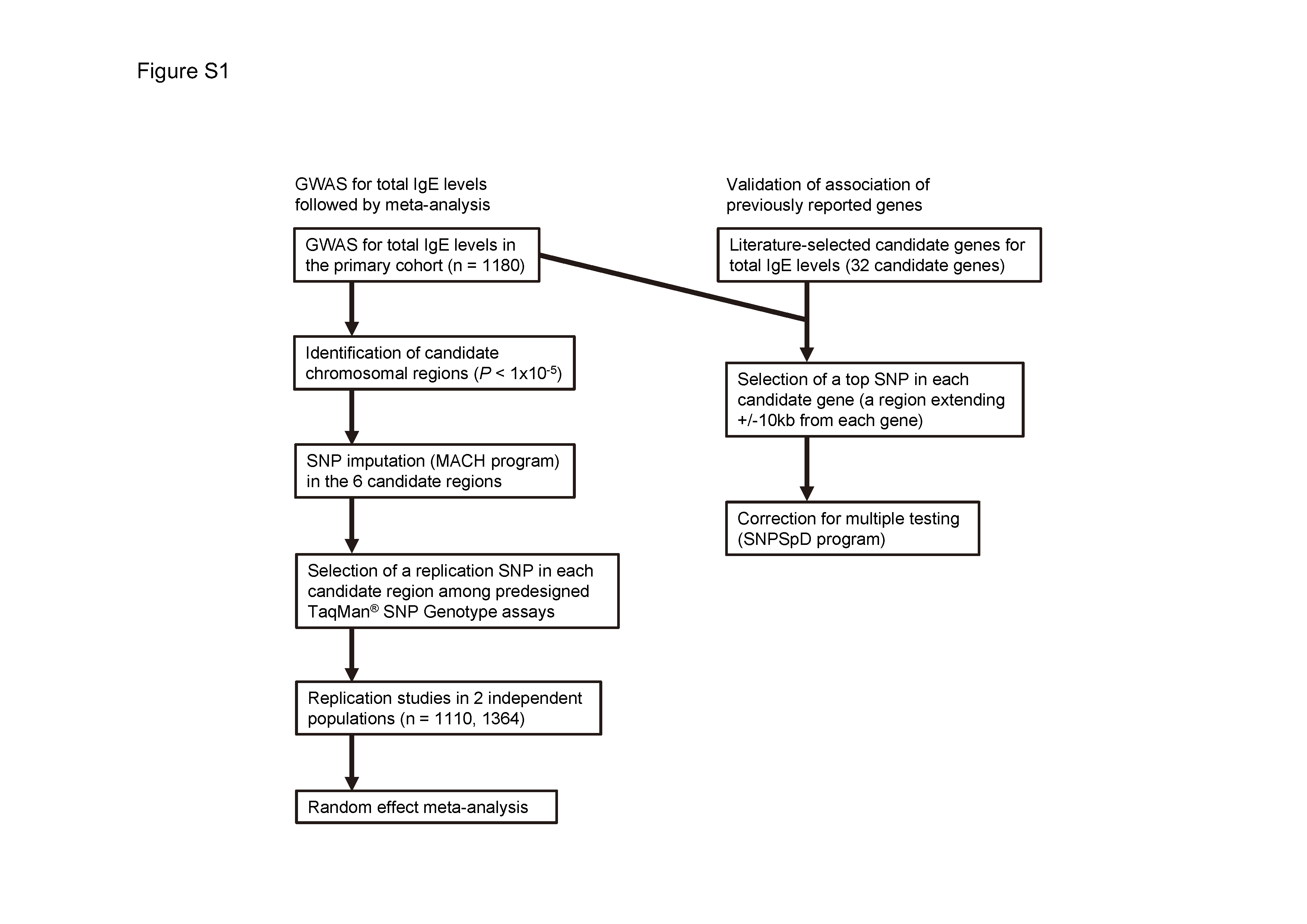

Supplement: Figure S1 — Study flow chart. GWAS for total IgE levels was performed, followed by replication studies and meta-analysis. Validation of previously reported genes for IgE was also conducted using the GWAS data. (TIFF) [file pone.0080941.s001.tiff]

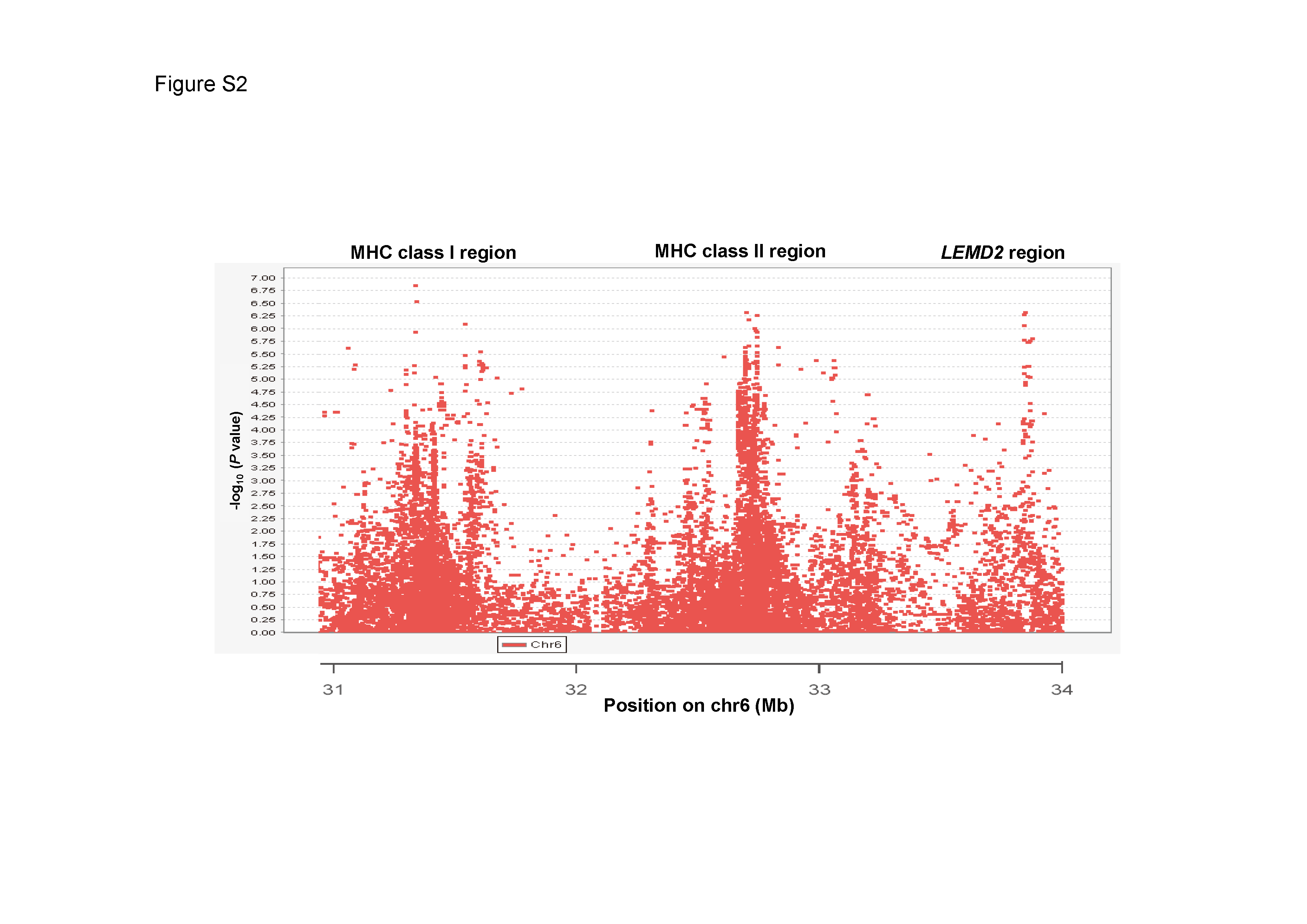

Supplement: Figure S2 — Fine-mapping association plots on chromosome 6p21. Three peaks are identified: the MHC class I, MHC class II, and LEMD2 regions. (TIFF) [file pone.0080941.s002.tiff]

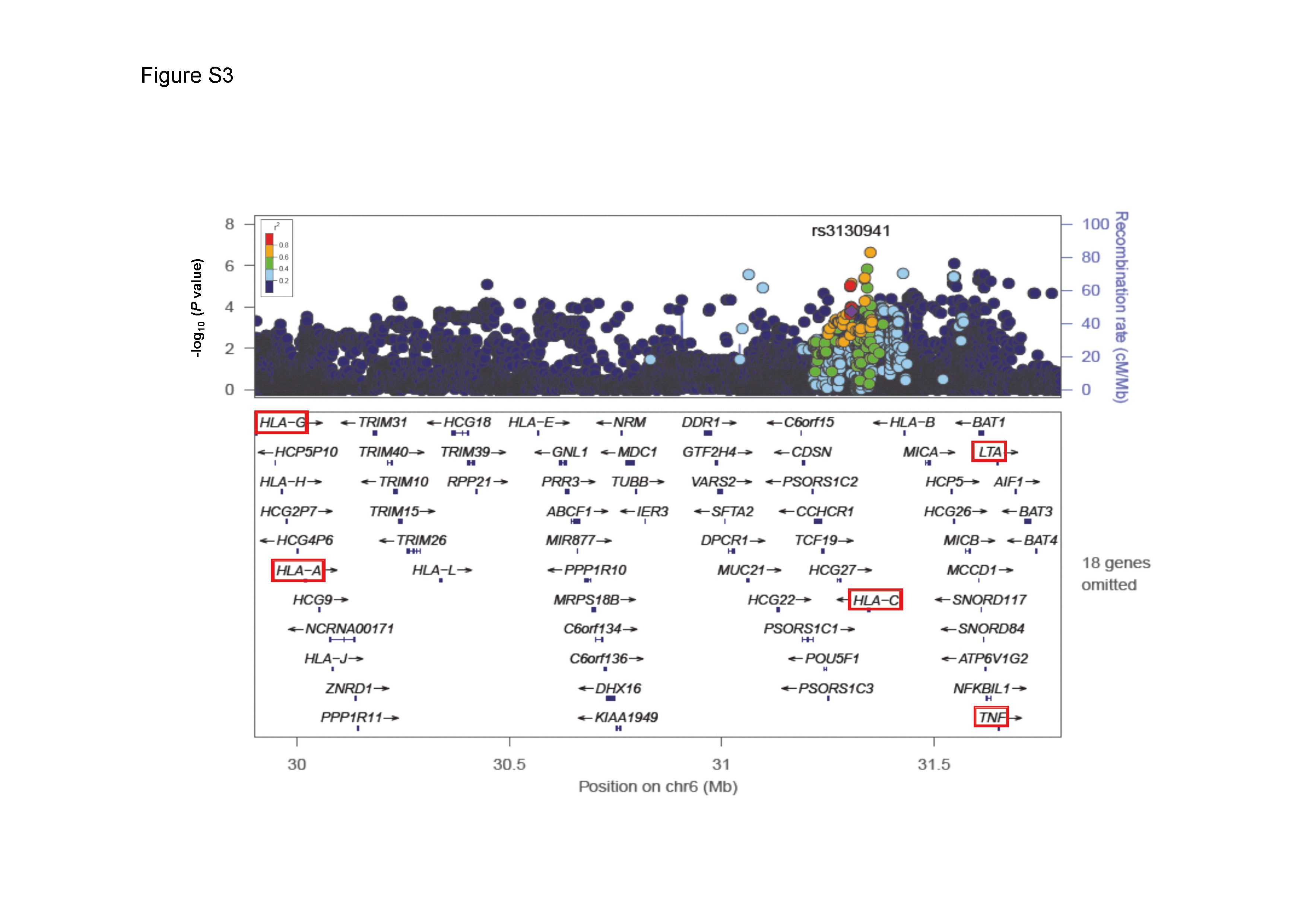

Supplement: Figure S3 — Fine-mapping association plots in the MHC class I region. The color of each circle reflects the LD (r2) between a particular SNP and rs3130941 indicated as a purple diamond. (TIFF) [file pone.0080941.s003.tiff]
