# Supplementary material for: Genome-Wide Association Study for Levels of Total Serum IgE Identifies HLA-C in a Japanese Population
Source: PLoS One. 2013 Dec 4;8(12):e80941. doi: 10.1371/journal.pone.0080941 (PMC3851760; doi:10.1371/journal.pone.0080941)
Supplement: Table S1 — Results of meta-analysis for nonasthmatic healthy individuals only. (DOCX) [file pone.0080941.s004.docx]

**Table S1.** Results of meta-analysis for nonasthmatic healthy individuals only.

|  |  | **Tsukuba cohort** | | | | **Hokkaido cohort** | **Fukui Cohort** | **Meta-analysis** |
| --- | --- | --- | --- | --- | --- | --- | --- | --- |
|  |  | **(n = 967)** | | | | **(n = 619)** | **(n = 1275)** | **(n = 2861)** |
| **Chromosome** | **Gene** | **SNP for replication study** | **Minor allele** | ***P* value** | **β** | ***P* value** | ***P* value** | ***P* value** |
| 1q23.1 | *PYHIN1/IFI16* | rs3754466 | C | 7.38E-05 | -0.128 | 0.207 | 0.206 | 0.365 |
| 6p21.3 | MHC class I | rs3130941 | C | 9.80E-04 | 0.095 | 1.06.E-04 | 2.19.E-05 | 1.30E-10 |
| 6p21.3 | MHC class II | rs28366296 | A | 7.90E-05 | -0.100 | 0.207 | 1.68.E-02 | 7.73E-06 |
| 6p21.31 | *LEMD2* | rs943474 | G | 2.64E-05 | 0.146 | 4.29E-02 | 0.242 | 1.21E-02 |
| 11q24.1 | *GRAMD1B* | rs7939777 | C | 2.22E-06 | -0.121 | 2.43E-02^*^ | 2.94.E-03 | 0.451 |
| 13q21.31 | none | rs3106598 | G | 5.26E-05 | 0.101 | 0.540 | 0.882 | 0.354 |

^*^The direction of the effect was opposite to that of the Tsukuba cohort.
