# Supplementary material for: Genome-Wide Association Study for Levels of Total Serum IgE Identifies HLA-C in a Japanese Population
Source: PLoS One. 2013 Dec 4;8(12):e80941. doi: 10.1371/journal.pone.0080941 (PMC3851760; doi:10.1371/journal.pone.0080941)
Supplement: Table S2 — Results of meta-analysis after inclusion of atopic status as a covariate. (DOCX) [file pone.0080941.s005.docx]

**Table S2.** Results of meta-analysis after inclusion of atopic status as a covariate.

|  |  | **Tsukuba cohort** | | | | **Hokkaido cohort** | **Fukui Cohort** | **Meta-analysis** |
| --- | --- | --- | --- | --- | --- | --- | --- | --- |
| **Chromosome** | **Gene** | **SNP for replication study** | **Minor allele** | ***P* value** | **β** | ***P* value** | ***P* value** | ***P* value** |
| 1q23.1 | *PYHIN1/IFI16* | rs3754466 | C | 5.71E-04 | -0.094 | 0.268 | 0.308 | 0.352 |
| 6p21.3 | MHC class I | rs3130941 | C | 1.72E-05 | 0.104 | 2.36E-02 | 5.76E-05 | 2.11E-09 |
| 6p21.3 | MHC class II | rs28366296 | A | 2.10E-04 | -0.080 | 0.106 | 1.50E-03 | 6.86E-07 |
| 6p21.31 | *LEMD2* | rs943474 | G | 2.39E-05 | 0.125 | 0.192 | 2.08E-02 | 7.48E-04 |
| 11q24.1 | *GRAMD1B* | rs7939777 | C | 7.88E-05 | -0.085 | 0.282^*^ | 8.66E-04 | 0.170 |
| 13q21.31 | none | rs3106598 | G | 9.91E-06 | 0.093 | 0.583 | 0.313 | 0.539 |

^*^The direction of the effect was opposite to that of the Tsukuba cohort.
