# Supplementary material for: Genome-Wide Association Study for Levels of Total Serum IgE Identifies HLA-C in a Japanese Population
Source: PLoS One. 2013 Dec 4;8(12):e80941. doi: 10.1371/journal.pone.0080941 (PMC3851760; doi:10.1371/journal.pone.0080941)
Supplement: Table S3 — Previously reported polymorphisms significantly associated with total serum IgE. (DOCX) [file pone.0080941.s006.docx]

**Table S3.** Previously reported polymorphisms significantly associated with total serum IgE.

| Gene | Polymorphism | *P* value previously reported | *P* value in our GWAS^*^ | Reference |
| --- | --- | --- | --- | --- |
| *ADAM33* | rs2280091 | 0.0033 | NA | [1] |
|  | rs597980 | 0.032 | NA | [2] |
|  | rs574174 | 0.01 | NA | [3] |
|  | rs528557 | 0.041 | NA | [4] |
| *ADRB2* | rs1042714 | <0.0001 | 0.846 | [5] |
|  | rs1042713 | 0.04 | 0.475 | [6] |
| *CCL11* | rs16969415 | 0.0006 | NA | [7] |
|  | rs3744508 | 0.002 | NA | [8] |
|  | rs17809012 | 0.016 | NA | [9] |
| *CD14* | rs2569190 | 0.0016 | NA | [10] |
|  | rs3138078 | 0.034 | NA | [11] |
|  | rs2563310 | 0.034 | NA | [12] |
| *CMA1* | rs1800875 | 0.0004 | NA | [13] |
| *CTLA4* | rs231775 | 0.0005 | NA | [14] |
|  | rs5472909 | 0.0047 | NA | [15] |
| *DARC* | rs13962 | 2.21E-11 | NA | [16]^‡^ |
|  | rs2814778 | 0.017 | NA | [17] |
| *FCER1A* | rs2251746 | 1.85E-20 | 0.515 | [18]^‡^ |
|  | rs4656784 | 1.68E-16 | NA | [16]^‡^ |
|  | rs2427827 | 0.001 | NA | [19] |
|  | rs2427837 | 0.014 | NA | [20] |
| *FLG* | 2282del4 | 0.009 | NA | [21] |
|  | rs11584340 | <0.05 | NA | [22] |
| *GSTP1* | rs1695 | 0.02 | 0.911 | [23] |
| *HLA-A* | rs2517754 | 3.61E-09 | NA | [16]^‡^ |
| *HLA-C* | rs3130941 | 1.07E-10 | NA | Current study^‡^ |
| *HLA-DQA2* | rs2858331 | 1.44E-08 | 0.000217 | [16]^‡^ |
| *HLA-DQB1* | DQB1*0301 | 0.008 | NA | [24] |
|  | rs1063355 | 0.01 | 0.000216 | [25] |
| *HLA-DRB1* | rs9271300 | 8.3E-15 | 0.0813 | [26]^‡^ |
|  | DRB1*07 | ＜0.0001 | NA | [27] |
|  | DRB1*01 | ＜0.004 | NA | [28] |
|  | DRB1*14 | 0.02 | NA | [24] |
| *HLA-G* | rs2523809 | 4.34E-08 | 0.707 | [16]^‡^ |
| *IFNG* | (CA)_n_ intron 1 | 0.0001 | NA | [29] |
|  | rs2430561 | <0.001 | NA | [30] |
| *IL4* | rs2070874 | 0.0001 | NA | [31] |
|  | rs2243290 | 0.0005 | 0.00342^†^ | [32] |
|  | rs2243250 | 0.001 | NA | [33] |
| *IL4R* | rs1805010 | 0.0021 | NA | [34] |
|  | rs1801275 | 0.02 | 0.948 | [35] |
| *IL10* | rs1800872 | <0.001 | NA | [36] |
| *IL13* | rs20541 | 0.00002 | 0.0819 | [37] |
|  | rs1881457 | 0.00002 | NA | [38] |
|  | rs1295685 | <0.0001 | NA | [39] |
|  | rs848 | <0.0001 | NA | [39] |
|  | rs1295686 | 0.00022 | 0.0741 | [40] |
|  | rs1800925 | 0.0002 | NA | [41] |
| *LTA* | rs909253 | 0.0032 | NA | [42] |
| *MS4A2* | rs1441586 | 0.0004 | NA | [43] |
|  | rs569108 | <0.001 | NA | [44] |
|  | Rsa I-In2 | 0.04 | NA | [24] |
| *NOD2* | rs2066845 | 0.006 | NA | [45] |
|  | rs5743293 | 0.009 | NA | [46] |
| *NOS1* | rs2682826 | <0.01 | 0.320 | [47] |
|  | (AAT)_n_ intron 20 | 0.015 | NA | [48] |
| *NPSR1* | rs323922 | 0.004 | NA | [49] |
|  | rs740347 | 0.001 | NA | [50] |
|  | SNP546333 | 0.015 | NA | [51] |
| *RAD50* | rs2040704 | 4.46E-08 | NA | [18]^‡^ |
| *SOBP* | rs7751374 | 3.68E-08 | 0.242 | [52]^‡^ |
| *STAT6* | rs1059513 | 1.99E-12 | 0.658 | [16]^‡^ |
|  | rs12368672 | 1.52E-05 | NA | [18]^‡^ |
|  | rs11172106 | 0.004 | NA | [53] |
|  | 1570C/T | 0.007 | NA | [54] |
|  | (GT)_n_ exon 1 | <0.01 | NA | [55] |
|  | rs324011 | 0.015 | NA | [56] |
|  | rs324015 | 0.042 | 0.348 | [57] |
| *TBX18* | rs10944017 | 3.88E-08 | NA | [52]^‡^ |
| *TLR2* | rs4696480 | 0.01 | NA | [58] |
|  | rs5743708 | 0.04 | NA | [59] |
| *TNF* | rs1800630 | 0.0024 | NA | [60] |
|  | rs361525 | <0.05 | NA | [61] |
|  | rs1800629 | <0.05 | NA | [61] |

The most significant *P* value in each study is shown.

^*^*P* value in our primary GWAS. ^‡^GWAS. ^†^The direction of the effect was opposite.

NA = not applicable.

**Table S3 references**

1. Howard TD, Postma DS, Jongepier H, Moore WC, Koppelman GH, et al. (2003) Association of a disintegrin and metalloprotease 33 (ADAM33) gene with asthma in ethnically diverse populations. J Allergy Clin Immunol 112: 717-722.

2. Werner M, Herbon N, Gohlke H, Altmuller J, Knapp M, et al. (2004) Asthma is associated with single-nucleotide polymorphisms in ADAM33. Clin Exp Allergy 34: 26-31.

3. Vergara CI, Acevedo N, Jimenez S, Martinez B, Mercado D, et al. (2010) A Six-SNP haplotype of ADAM33 is associated with asthma in a population of Cartagena, Colombia. Int Arch Allergy Immunol 152: 32-40.

4. Yang KD, Chang JC, Chuang H, Liang HM, Kuo HC, et al. (2010) Gene-gene and gene-environment interactions on IgE production in prenatal stage. Allergy 65: 731-739.

5. Qiu YY, Zhang XL, Yin KS (2006) Association between beta2-adrenergic receptor genetic polymorphisms and total serum IgE in asthmatic patients of Chinese Han nationality. Respiration 73: 180-184.

6. Giubergia V, Zelazko M, Roy A, Gravina LP, Gonzalez Pena H, et al. (2009) Beta 2-adrenergic polymorphisms and total serum IgE levels in children with asthma from Argentina. Ann Allergy Asthma Immunol 102: 308-313.

7. Raby BA, Van Steen K, Lazarus R, Celedon JC, Silverman EK, et al. (2006) Eotaxin polymorphisms and serum total IgE levels in children with asthma. J Allergy Clin Immunol 117: 298-305.

8. Shin HD, Kim LH, Park BL, Jung JH, Kim JY, et al. (2003) Association of Eotaxin gene family with asthma and serum total IgE. Hum Mol Genet 12: 1279-1285.

9. Batra J, Rajpoot R, Ahluwalia J, Devarapu SK, Sharma SK, et al. (2007) A hexanucleotide repeat upstream of eotaxin gene promoter is associated with asthma, serum total IgE and plasma eotaxin levels. J Med Genet 44: 397-403.

10. Sharma M, Batra J, Mabalirajan U, Goswami S, Ganguly D, et al. (2004) Suggestive evidence of association of C-159T functional polymorphism of the CD14 gene with atopic asthma in northern and northwestern Indian populations. Immunogenetics 56: 544-547.

11. Tan CY, Chen YL, Wu LS, Liu CF, Chang WT, et al. (2006) Association of CD14 promoter polymorphisms and soluble CD14 levels in mite allergen sensitization of children in Taiwan. J Hum Genet 51: 59-67.

12. Wang JY, Wang LM, Lin CG, Chang AC, Wu LS (2005) Association study using combination analysis of SNP and STRP markers: CD14 promoter polymorphism and IgE level in Taiwanese asthma children. J Hum Genet 50: 36-41.

13. Sharma S, Rajan UM, Kumar A, Soni A, Ghosh B (2005) A novel (TG)n(GA)m repeat polymorphism 254 bp downstream of the mast cell chymase (CMA1) gene is associated with atopic asthma and total serum IgE levels. J Hum Genet 50: 276-282.

14. Howard TD, Postma DS, Hawkins GA, Koppelman GH, Zheng SL, et al. (2002) Fine mapping of an IgE-controlling gene on chromosome 2q: Analysis of CTLA4 and CD28. J Allergy Clin Immunol 110: 743-751.

15. Hizawa N, Yamaguchi E, Jinushi E, Konno S, Kawakami Y, et al. (2001) Increased total serum IgE levels in patients with asthma and promoter polymorphisms at CTLA4 and FCER1B. J Allergy Clin Immunol 108: 74-79.

16. Granada M, Wilk JB, Tuzova M, Strachan DP, Weidinger S, et al. (2012) A genome-wide association study of plasma total IgE concentrations in the Framingham Heart Study. J Allergy Clin Immunol 129: 840-845 e821.

17. Vergara C, Tsai YJ, Grant AV, Rafaels N, Gao L, et al. (2008) Gene encoding Duffy antigen/receptor for chemokines is associated with asthma and IgE in three populations. Am J Respir Crit Care Med 178: 1017-1022.

18. Weidinger S, Gieger C, Rodriguez E, Baurecht H, Mempel M, et al. (2008) Genome-wide scan on total serum IgE levels identifies FCER1A as novel susceptibility locus. PLoS Genet 4: e1000166.

19. Potaczek DP, Owczarek D, Okumura K, Mach T, Undas A, et al. (2010) An association between functional FceRIalpha polymorphisms and total serum IgE levels in patients with inflammatory bowel disease. Scand J Gastroenterol 45: 766-767.

20. Zhou J, Zhou Y, Lin LH, Wang J, Peng X, et al. (2012) Association of polymorphisms in the promoter region of FCER1A gene with atopic dermatitis, chronic uticaria, asthma, and serum immunoglobulin E levels in a Han Chinese population. Hum Immunol 73: 301-305.

21. Rogers AJ, Celedon JC, Lasky-Su JA, Weiss ST, Raby BA (2007) Filaggrin mutations confer susceptibility to atopic dermatitis but not to asthma. J Allergy Clin Immunol 120: 1332-1337.

22. Wang IJ, Lin TJ, Kuo CF, Lin SL, Lee YL, et al. (2011) Filaggrin polymorphism P478S, IgE level, and atopic phenotypes. Br J Dermatol 164: 791-796.

23. Gerbase MW, Keidel D, Imboden M, Gemperli A, Bircher A, et al. (2011) Effect modification of immunoglobulin E-mediated atopy and rhinitis by glutathione S-transferase genotypes in passive smokers. Clin Exp Allergy 41: 1579-1586.

24. Cardaba B, Moffatt MF, Fernandez E, Jurado A, Rojo M, et al. (2001) Allergy to dermatophagoides in a group of Spanish gypsies: genetic restrictions. Int Arch Allergy Immunol 125: 297-306.

25. Li X, Howard TD, Zheng SL, Haselkorn T, Peters SP, et al. (2010) Genome-wide association study of asthma identifies RAD50-IL13 and HLA-DR/DQ regions. J Allergy Clin Immunol 125: 328-335.

26. Moffatt MF, Gut IG, Demenais F, Strachan DP, Bouzigon E, et al. (2010) A large-scale, consortium-based genomewide association study of asthma. N Engl J Med 363: 1211-1221.

27. Moffatt MF, Faux JA, Lester S, Pare P, McCluskey J, et al. (2003) Atopy, respiratory function and HLA-DR in Aboriginal Australians. Hum Mol Genet 12: 625-630.

28. Woszczek G, Kowalski ML, Borowiec M (2002) Association of asthma and total IgE levels with human leucocyte antigen-DR in patients with grass allergy. Eur Respir J 20: 79-85.

29. Nagarkatti R, C BR, Rishi JP, Chetiwal R, Shandilya V, et al. (2002) Association of IFNG gene polymorphism with asthma in the Indian population. J Allergy Clin Immunol 110: 410-412.

30. Hussein YM, Ahmad AS, Ibrahem MM, El Tarhouny SA, Shalaby SM, et al. (2009) Interferon gamma gene polymorphism as a biochemical marker in Egyptian atopic patients. J Investig Allergol Clin Immunol 19: 292-298.

31. Dmitrieva-Zdorova EV, Voronko OE, Latysheva EA, Storozhakov GI, Archakov AI (2012) Analysis of polymorphisms in T(H)2-associated genes in Russian patients with atopic bronchial asthma. J Investig Allergol Clin Immunol 22: 126-132.

32. Basehore MJ, Howard TD, Lange LA, Moore WC, Hawkins GA, et al. (2004) A comprehensive evaluation of IL4 variants in ethnically diverse populations: association of total serum IgE levels and asthma in white subjects. J Allergy Clin Immunol 114: 80-87.

33. Lu MP, Chen RX, Wang ML, Zhu XJ, Zhu LP, et al. (2011) Association study on IL4, IL13 and IL4RA polymorphisms in mite-sensitized persistent allergic rhinitis in a Chinese population. PLoS One 6: e27363.

34. Gueant-Rodriguez RM, Romano A, Beri-Dexheimer M, Viola M, Gaeta F, et al. (2006) Gene-gene interactions of IL13 and IL4RA variants in immediate allergic reactions to betalactam antibiotics. Pharmacogenet Genomics 16: 713-719.

35. Cornejo-Garcia JA, Gueant-Rodriguez RM, Torres MJ, Blanca-Lopez N, Tramoy D, et al. (2012) Biological and genetic determinants of atopy are predictors of immediate-type allergy to betalactams, in Spain. Allergy 67: 1181-1185.

36. Negoro T, Orihara K, Irahara T, Nishiyama H, Hagiwara K, et al. (2006) Influence of SNPs in cytokine-related genes on the severity of food allergy and atopic eczema in children. Pediatr Allergy Immunol 17: 583-590.

37. Graves PE, Kabesch M, Halonen M, Holberg CJ, Baldini M, et al. (2000) A cluster of seven tightly linked polymorphisms in the IL-13 gene is associated with total serum IgE levels in three populations of white children. J Allergy Clin Immunol 105: 506-513.

38. Maier LM, Howson JM, Walker N, Spickett GP, Jones RW, et al. (2006) Association of IL13 with total IgE: evidence against an inverse association of atopy and diabetes. J Allergy Clin Immunol 117: 1306-1313.

39. Genuneit J, Cantelmo JL, Weinmayr G, Wong GW, Cooper PJ, et al. (2009) A multi-centre study of candidate genes for wheeze and allergy: the International Study of Asthma and Allergies in Childhood Phase 2. Clin Exp Allergy 39: 1875-1888.

40. Donfack J, Schneider DH, Tan Z, Kurz T, Dubchak I, et al. (2005) Variation in conserved non-coding sequences on chromosome 5q and susceptibility to asthma and atopy. Respir Res 6: 145.

41. Liu X, Beaty TH, Deindl P, Huang SK, Lau S, et al. (2003) Associations between total serum IgE levels and the 6 potentially functional variants within the genes IL4, IL13, and IL4RA in German children: the German Multicenter Atopy Study. J Allergy Clin Immunol 112: 382-388.

42. Trabetti E, Patuzzo C, Malerba G, Galavotti R, Martinati LC, et al. (1999) Association of a lymphotoxin alpha gene polymorphism and atopy in Italian families. J Med Genet 36: 323-325.

43. Hizawa N, Yamaguchi E, Jinushi E, Kawakami Y (2000) A common FCER1B gene promoter polymorphism influences total serum IgE levels in a Japanese population. Am J Respir Crit Care Med 161: 906-909.

44. Nagata H, Mutoh H, Kumahara K, Arimoto Y, Tomemori T, et al. (2001) Association between nasal allergy and a coding variant of the Fc epsilon RI beta gene Glu237Gly in a Japanese population. Hum Genet 109: 262-266.

45. Weidinger S, Klopp N, Rummler L, Wagenpfeil S, Baurecht HJ, et al. (2005) Association of CARD15 polymorphisms with atopy-related traits in a population-based cohort of Caucasian adults. Clin Exp Allergy 35: 866-872.

46. Kabesch M, Peters W, Carr D, Leupold W, Weiland SK, et al. (2003) Association between polymorphisms in caspase recruitment domain containing protein 15 and allergy in two German populations. J Allergy Clin Immunol 111: 813-817.

47. Holla LI, Schuller M, Buckova D, Vacha J (2004) Neuronal nitric oxide synthase gene polymorphism and IgE-mediated allergy in the Central European population. Allergy 59: 548-552.

48. Wang TN, Tseng HI, Kao CC, Chu YT, Chen WY, et al. (2010) The effects of NOS1 gene on asthma and total IgE levels in Taiwanese children, and the interactions with environmental factors. Pediatr Allergy Immunol 21: 1064-1071.

49. Laitinen T, Polvi A, Rydman P, Vendelin J, Pulkkinen V, et al. (2004) Characterization of a common susceptibility locus for asthma-related traits. Science 304: 300-304.

50. Vergara C, Jimenez S, Acevedo N, Martinez B, Mercado D, et al. (2009) Association of G-protein-coupled receptor 154 with asthma and total IgE in a population of the Caribbean coast of Colombia. Clin Exp Allergy 39: 1558-1568.

51. Kormann MS, Carr D, Klopp N, Illig T, Leupold W, et al. (2005) G-Protein-coupled receptor polymorphisms are associated with asthma in a large German population. Am J Respir Crit Care Med 171: 1358-1362.

52. Levin AM, Mathias RA, Huang L, Roth LA, Daley D, et al. (2012) A meta-analysis of genome-wide association studies for serum total IgE in diverse study populations. J Allergy Clin Immunol.

53. Hong X, Tsai HJ, Liu X, Arguelles L, Kumar R, et al. (2010) Does genetic regulation of IgE begin in utero? Evidence from T(H)1/T(H)2 gene polymorphisms and cord blood total IgE. J Allergy Clin Immunol 126: 1059-1067, 1067 e1051.

54. Duetsch G, Illig T, Loesgen S, Rohde K, Klopp N, et al. (2002) STAT6 as an asthma candidate gene: polymorphism-screening, association and haplotype analysis in a Caucasian sib-pair study. Hum Mol Genet 11: 613-621.

55. Yabiku K, Hayashi M, Komiya I, Yamada T, Kinjo Y, et al. (2007) Polymorphisms of interleukin (IL)-4 receptor alpha and signal transducer and activator of transcription-6 (Stat6) are associated with increased IL-4Ralpha-Stat6 signalling in lymphocytes and elevated serum IgE in patients with Graves' disease. Clin Exp Immunol 148: 425-431.

56. Weidinger S, Klopp N, Wagenpfeil S, Rummler L, Schedel M, et al. (2004) Association of a STAT 6 haplotype with elevated serum IgE levels in a population based cohort of white adults. J Med Genet 41: 658-663.

57. Zhang G, Candelaria P, Makela JM, Khoo SK, Hayden MC, et al. (2011) Disparity of innate immunity-related gene effects on asthma and allergy on Karelia. Pediatr Allergy Immunol 22: 621-630.

58. Bottema RW, Kerkhof M, Reijmerink NE, Thijs C, Smit HA, et al. (2010) Gene-gene interaction in regulatory T-cell function in atopy and asthma development in childhood. J Allergy Clin Immunol 126: 338-346, 346 e331-310.

59. Kormann MS, Ferstl R, Depner M, Klopp N, Spiller S, et al. (2009) Rare TLR2 mutations reduce TLR2 receptor function and can increase atopy risk. Allergy 64: 636-642.

60. Sharma S, Sharma A, Kumar S, Sharma SK, Ghosh B (2006) Association of TNF haplotypes with asthma, serum IgE levels, and correlation with serum TNF-alpha levels. Am J Respir Cell Mol Biol 35: 488-495.

61. Krasznai M, Szaniszlo K, Kraxner H, Vargha E, Kovacs M, et al. (2011) Association of TLR-4 and TNF-alpha polymorphisms with clinical symptoms and cytokine levels in patients with allergic rhinitis. Eur Arch Otorhinolaryngol 268: 561-567.
