# Supplementary material for: Genome-Wide Association Study for Levels of Total Serum IgE Identifies HLA-C in a Japanese Population
Source: PLoS One. 2013 Dec 4;8(12):e80941. doi: 10.1371/journal.pone.0080941 (PMC3851760; doi:10.1371/journal.pone.0080941)
Supplement: Table S4 — Genetic influences of SNPs in the MHC class I/II regions on the association between rs3130941 and total IgE levels. (DOCX) [file pone.0080941.s007.docx]

**Table S4.** Genetic influences of SNPs in the MHC class I/II regions on the association between rs3130941and total IgE levels.

| **SNP** | **LD** **(r^2^) with rs3130941** | ***P* value adjusted for each SNP^*^** |
| --- | --- | --- |
| rs2517754 (*HLA-A*) | 0.008 | 3.34E-04 |
| rs2571391 (*HLA-A*) | 0.047 | 2.27E-03 |
| rs2523809 (*HLA-G*) | 0.020 | 3.79E-04 |
| rs909253 (*LTA*) | 0.100 | 6.07E-04 |
| rs1800629 (*TNF*) | 0.002 | 2.57E-04 |
| rs361525 (*TNF*) | NA | NA |
| rs1800630 (*TNF*) | 0.017 | 4.86E-04 |
| rs28366296 (MHC class II) | 0.064 | 5.51E-03 |

NA = not applicable; LD = linkage disequilibrium.

^*^*P* values given for the association between rs3130941 and total IgE levels in our primary cohort.

When none of these SNPs were included in the statistical model, the *P* value for rs3130941 was 2.28x10^-4^.
